# Epidemiological and Genetic Characteristics of Clinical Carbapenem-Resistant *Pseudomonas aeruginosa* Strains in Guangdong Province, China

**DOI:** 10.1128/spectrum.04261-22

**Published:** 2023-04-20

**Authors:** Yonggang Zhao, Dingqiang Chen, Kaichao Chen, Miaomiao Xie, Jiubiao Guo, Edward Wai Chi Chan, Lu Xie, Jingbo Wang, Enqi Chen, Sheng Chen, Weijun Chen, Lars Jelsbak

**Affiliations:** a Department of Biotechnology and Biomedicine, Technical University of Denmark, Lyngby, Denmark; b Microbiome Medicine Center, Department of Laboratory Medicine, Zhujiang Hospital, Southern Medical University, Guangzhou, People’s Republic of China; c Department of Infectious Diseases and Public Health, Jockey Club College of Veterinary Medicine and Life Sciences, City University of Hong Kong, Kowloon, People’s Republic of China; d College of Pharmacy-Shenzhen Technology University, Shenzhen, People’s Republic of China; e Research Center for Micro-Ecological Agent Engineering and Technology of Guangdong Province, Guangzhou, People’s Republic of China; f BGI-Shenzhen, Shenzhen, People’s Republic of China; Emory University School of Medicine

**Keywords:** *Pseudomonas aeruginosa*, carbapenem resistance, *oprD*, efflux-pump-encoding genes, molecular epidemiology

## Abstract

Carbapenem-resistant Pseudomonas aeruginosa (CRPA) is a bacterial pathogen that may cause serious drug-resistant infections that are potentially fatal. To investigate the genetic characteristics of these organisms, we tested 416 P. aeruginosa strains recovered from 12 types of clinical samples collected in 29 different hospital wards in 10 hospitals in Guangdong Province, China, from 2017 to 2020. These strains were found to belong to 149 known sequence types (STs) and 72 novel STs, indicating that transmission of these strains involved multiple routes. A high rate of resistance to imipenem (89.4%) and meropenem (79.4%) and a high prevalence of pathogenic serotypes (76.4%) were observed among these strains. Six STs of global high-risk clones (HiRiCs) and a novel HiRiC strains, ST1971, which exhibited extensive drug resistance, were identified. Importantly, ST1971 HiRiC, which was unique in China, also exhibited high virulence, which alarmed the further surveillance on this highly virulent and highly resistant clone. Inactivation of the *oprD* gene and overexpression of efflux systems were found to be mainly responsible for carbapenem resistance in these strains; carriage of metallo-β-lactamase (MBL)-encoding genes was less common. Interestingly, frameshift mutations (49.0%) and introduction of a stop codon (22.4%) into the *oprD* genes were the major mechanisms of imipenem resistance. On the other hand, expression of the MexAB-OprM efflux pump and MBL-encoding genes were mechanisms of resistance in >70% of meropenem-resistant strains. The findings presented here provide insights into the development of effective strategies for control of worldwide dissemination of CRPA.

**IMPORTANCE** Carbapenem-resistant Pseudomonas aeruginosa (CRPA) is a major concern in clinical settings worldwide, yet few genetic and epidemiological studies on CRPA strains have been performed in China. Here, we sequence and analyze the genomes of 416 P. aeruginosa strains from hospitals in China to elucidate the genetic, phenotypic, and transmission characteristics of CRPA strains and to identify the molecular signatures responsible for the observed increase in the prevalence of CRPA infections in China. These findings may provide new insight into the development of effective strategies for worldwide control of CRPA and minimize the occurrence of untreatable infections in clinical settings.

## INTRODUCTION

Pseudomonas aeruginosa is an important Gram-negative agent that often cause severe infections in human (1). A recent World Health Organization (WHO) report highlighted the public health concern regarding this pathogen, since multiple-drug-resistant P. aeruginosa was found to be responsible for 51,000 cases of nosocomial infections and 400 deaths each year in the United States alone (2). P. aeruginosa is a leading causative agent of chronic lung infections among cancer and cystic fibrosis patients (3) and is responsible for >90% of respiratory failure cases (4). In addition, a recent systematic review showed that P. aeruginosa-mediated ventilator-associated pneumonia was associated with a high mortality rate ranging from approximately 40 to 70% (5). In China, surveillance of clinical P. aeruginosa strains is part of a strategic plan to control hospital infections that has been implemented annually for nearly two decades. Records of this program, known as the Chinese Antimicrobial Surveillance Network (CHINET), showed that the rate of resistance of P. aeruginosa strains to carbapenems in clinical settings remained relatively stable during from 2005 to 2018 (25 to 30%) and then dropped slightly in the past 3 years (2019 to 2021) to resistance rates of ~20 and ~24% to meropenem and imipenem, respectively.

P. aeruginosa is known to exhibit complex mechanisms of resistance to the carbapenems. To date, the principal resistance mechanism involves inactivation of the gene that codes for the porin OprD by mutations or mobile elements, reducing outer membrane permeability and impeding the entry of various antibiotics, including carbapenems (6). Overexpression of several efflux systems (MexAB-OprM, MexCD-OprJ, and MexXY-OprM) and hyperproduction of the chromosomal cephalosporinase AmpC are also closely related to mutation-mediated carbapenem resistance mechanisms (7). Different combinations of these mechanisms result in variable imipenem, meropenem, and doripenem MICs among the clinical strains (8). The situation was aggravated by the dissemination of carbapenemase-hydrolysis-encoding genes, including *bla*_GES_, *bla*_KPC_, *bla*_AIM_, *bla*_GIM_, *bla*_IMP_, *bla*_NDM_, *bla*_SPM_, *bla*_VIM_, and *bla*_OXA-198_ (9–11). Furthermore, transmission of carbapenem-resistant P. aeruginosa (CRPA) strains is also responsible for a sharp increase in the incidence of carbapenem resistance in recent years. Several P. aeruginosa strains that contain multidrug resistance (MDR)-encoding elements have undergone global transmission and are defined as high-risk clones (HiRiCs) (12), which acquired the ability to accumulate and switch resistance in response to environmental changes (13). At present, 10 HiRiC strains have been identified, most of which were found to acquire β-lactamase genes that encode extended-spectrum β-lactamases and carbapenem-hydrolyzing enzymes and exhibit resistance to either cephalosporin and carbapenem agents, respectively (14).

With the advent of sequencing technology, whole-genome sequencing (WGS) has been increasingly adopted for studying drug-resistant pathogens in health care systems (15). However, few genetic and epidemiological studies of CRPA strains have been performed in China. Here, we conducted a genome-based survey of the prevalence of CRPA strains collected from various wards in 10 hospitals in Guangdong Province, China. By investigating the molecular epidemiological, genetic, and phenotypic characteristics of strains that exhibit resistance to carbapenems, as well as the transmission characteristics of these strains, we were able to identify crucial factors responsible for causing an increase in prevalence of CRPA infections in China. Our findings provide significant insight into the development of effective strategies for the worldwide control of CRPA and will help to minimize the occurrence of untreatable infections in clinical settings.

## RESULTS

### Patient information.

**FIG 1:**
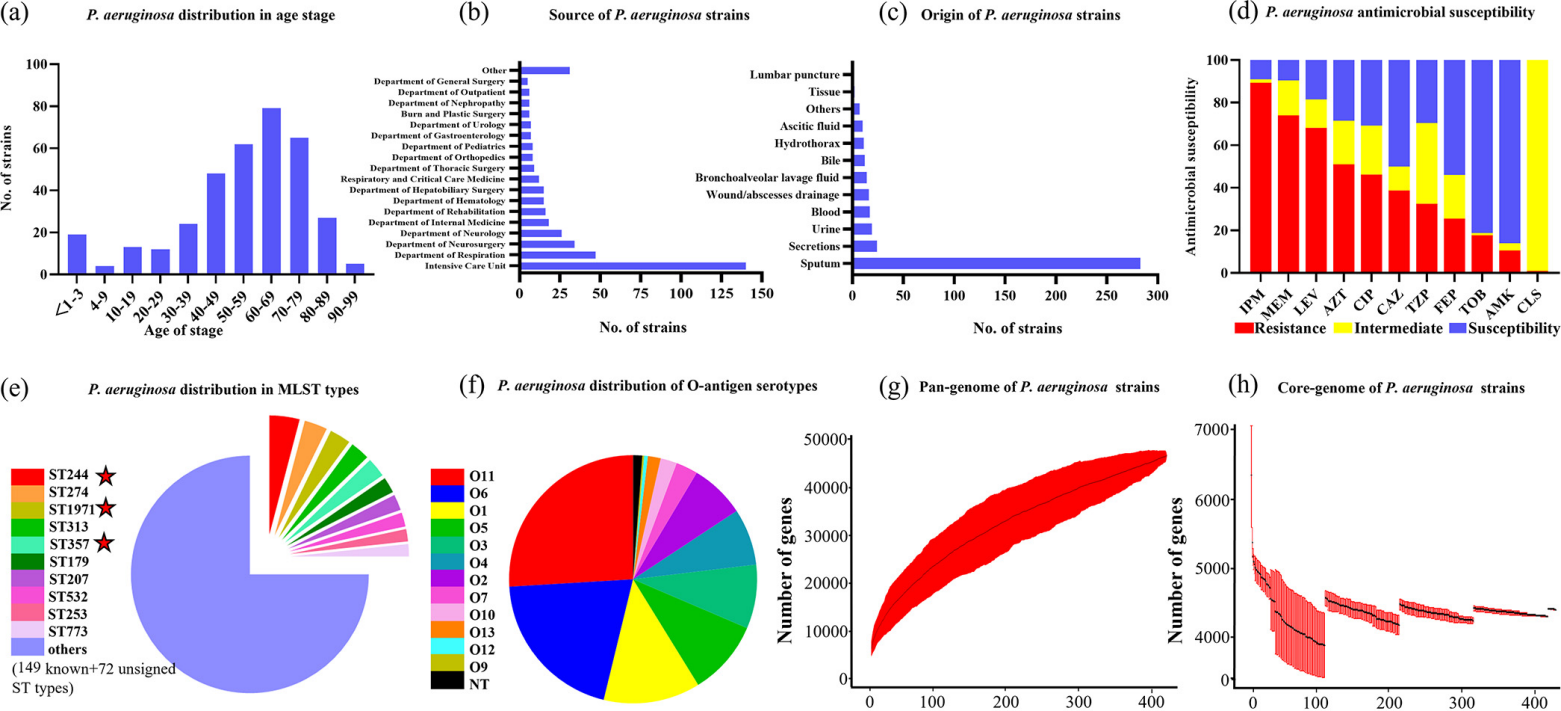
Phenotypic and epidemiological characteristics of 416 clinical P. aeruginosa strains collected from 10 tertiary hospitals in Guangdong, China, from 2017 to 2020. (a) Distribution of age of patients from whom the P. aeruginosa strains were recovered. (b) Rate of recovery of P. aeruginosa strains from different clinical departments. (c) Rate of recovery of P. aeruginosa strains from different clinical specimens. (d) Antimicrobial susceptibility of the 416 clinical P. aeruginosa strains. IMP, imipenem; MEM, meropenem; LEV, levofloxacin; AZT, aztreonam; CIP, ciprofloxacin; CAZ, ceftazidime; TZP, piperacillin-tazobactam; FEP, Cefepime; TOB, tobramycin; AMK, amikacin; CST, colistin. Interpretation of resistance phenotypes adheres to the CLSI M100-S26. (e and f) Distribution of the top 10 prevalent ST-types of strains (e) and serotypes (f). The five-pointed star indicates HiRiCs ST-types of strains. (g) Accumulation of pan genes. (h) Number of core genes (99% ≤ strains ≤ 100%) in the 416 P. aeruginosa strains.

A total of 416 P. aeruginosa strains collected from nonduplicate samples during the period from 30 November 2017 to 7 December 2020 were subjected to WGS. These strains were recovered from patients whose ages ranged from <1 to 99 years old, with 41.34% (*n *= 172) of the strains isolated from patients aged 65 years or older. The age distribution of patients from whom P. aeruginosa strains were recovered exhibited an increasing and then falling trend, with most of the strains being collected from patients of 60 to 69 years old (22.12%), followed by patients 70 to 79 years old (20.19%) (Fig. 1a). On the other hand, the proportion of strains collected from newborns was higher than that collected from the 4 to 9, 10 to 19, and 20 to 29 age groups, respectively. Nevertheless, 81.01% of these P. aeruginosa strains were isolated from patients aged 40 to 99.

A polychoric correlation test was performed to analyze the relationship between the age and antibiotic resistance level. The results showed that the increase in age of patients from whom samples were collected was associated with an increase in the MICs of piperacillin-tazobactam, ciprofloxacin, levofloxacin, amikacin, tobramycin, and cefepime (*P* < 0.05) (see Fig. S1 in the supplemental material), although the correlation rates are all <0.21. Compared to the high percentage of MDR strains of other age groups, half of the samples purified from patients ≤3 years old were susceptible strains (10/20, 50%), presumably due to low exposure to antibiotics. The proportion of P. aeruginosa strains collected from different wards and specimen types was also analyzed, with results showing that 33.7% of the strains were isolated from the intensive care unit (*n *= 140), followed by the departments of respiration (11.3%, *n *= 47) and neurosurgery (8.2%, *n *= 34). As many as 68.0% of the strains were purified from sputum, followed by secretions, urine, and blood, which accounted for 5.8, 4.6, and 4.1%, respectively, of the test strains (Fig. 1b and c).

### Antibiotic resistance spectrum of *P. aeruginosa* strains.

The antibiotic resistance profiles of the P. aeruginosa strains were determined. The P. aeruginosa strains were found to exhibit a high rate of resistance to imipenem (89.4%), meropenem (74.0%), and levofloxacin (68.2%), followed by aztreonam (51.1%), ciprofloxacin (46.3%), and ceftazidime (38.8%). The rates of susceptibility to cefepime, piperacillin-tazobactam, amikacin, and tobramycin were 54.0, 29.6, 86.0, and 81.20%, respectively. Almost all of the strains (99.0%) showed intermediate resistance to colistin (Fig. 1d). Detail information for the MICs is presented in Table 1. In addition, a total of 402 (96.6%) P. aeruginosa strains were found to belong to carbapenem-nonsusceptible P. aeruginosa which exhibited resistance to meropenem or imipenem or both. Multidrug resistance with resistance to over three categories of antibiotics was commonly observed among the test strains (64.8%); 15.7% of the strains were classified as extensively drug resistant (XDR), exhibiting resistance to more than six categories of antibiotics.

**TABLE 1:** Genetic characteristics and antimicrobial susceptibility of CRPA strains tested in this study

Antibiotics	No. of isolates at an MIC (mg/L)^*a*^ of:											Breakpoint(s)^*b*^	MIC (mg/L)		*R* (%)	Genetic resistance determinant(s)
													MIC_50_	MIC_90_		
	0.12	0.25	0.5	1	2	4	8	16	32	64	128					
Imipenem		3*		9	26	6	**34**	**337**†				≤2/≥8	≥16	≥16	89.40	Inactivation of *oprD*
Meropenem			4	19	17	67	**54**	**254**†				≤2/≥8	≥16	≥16	73.98	MexAB-OprM, mexCD-OprJ, *bla*_GES_, *bla*_KPC_, *bla*_AIM_, *bla*_GIM_, *bla*_IMP_, *bla*_SPM_, *bla*_VIM_, and *bla*_OXA-198_
Ceftazidime		1		2	129	15	61	46	**53**	**108**†		≤8/≥32	2	≥64	38.80	*bla*_AmpC_
Cefepime				8*	43	18	155	106	**85**†			≤8/≥32	8	≥32	25.54	Overexpression of MexCD-OprJ
Piperacillin-tazobactam						30*	57	36	107	50	**135**†	(≤16/4)/ (≥128/4)	32	≥128	32.53	*bla*_AmpC_
Colistin			390*	10	11	**4**						≤2/≥4	≤0.5	≤0.5	0.96	
Ciprofloxacin		69*	59	95	**76**	**116**†						≤0.5/≥2	1	≥4	46.27	*gyrA*^T83I^, *gyrA*^D87N^, *parE*^A473V^, *qnrVC*, *ampC*
Levofloxacin	2	2*	11	62	55	**130**	**153**†					≤1/≥4	4	≥8	68.19	MexXY-OprM (*mexZ*), MexVW-OprM
Tobramycin				315*	14	8	5	**73**†				≤4/≥16	≤1	≥16	17.59	MexXY-OprM
Amikacin					229*	67	29	32	14	**44**†		≤16/≥64	≤2	≥64	10.60	*aac*
Aztreonam					7	55	56	85	**61**	**151**†		≤8/≥32	32	≥64	51.08	MexAB-OprM

a Boldfacing indicates data for resistant strains. *, MIC ≤ the indicated value; †, MIC ≥ the indicated value.

b Breakpoint concentrations—expressed as “susceptibility (mg/L)/resistance (mg/L)”—are presented according to CLSI2022.

### WGS analysis of 416 clinical CRPA *P. aeruginosa* strains.

**FIG 2:**
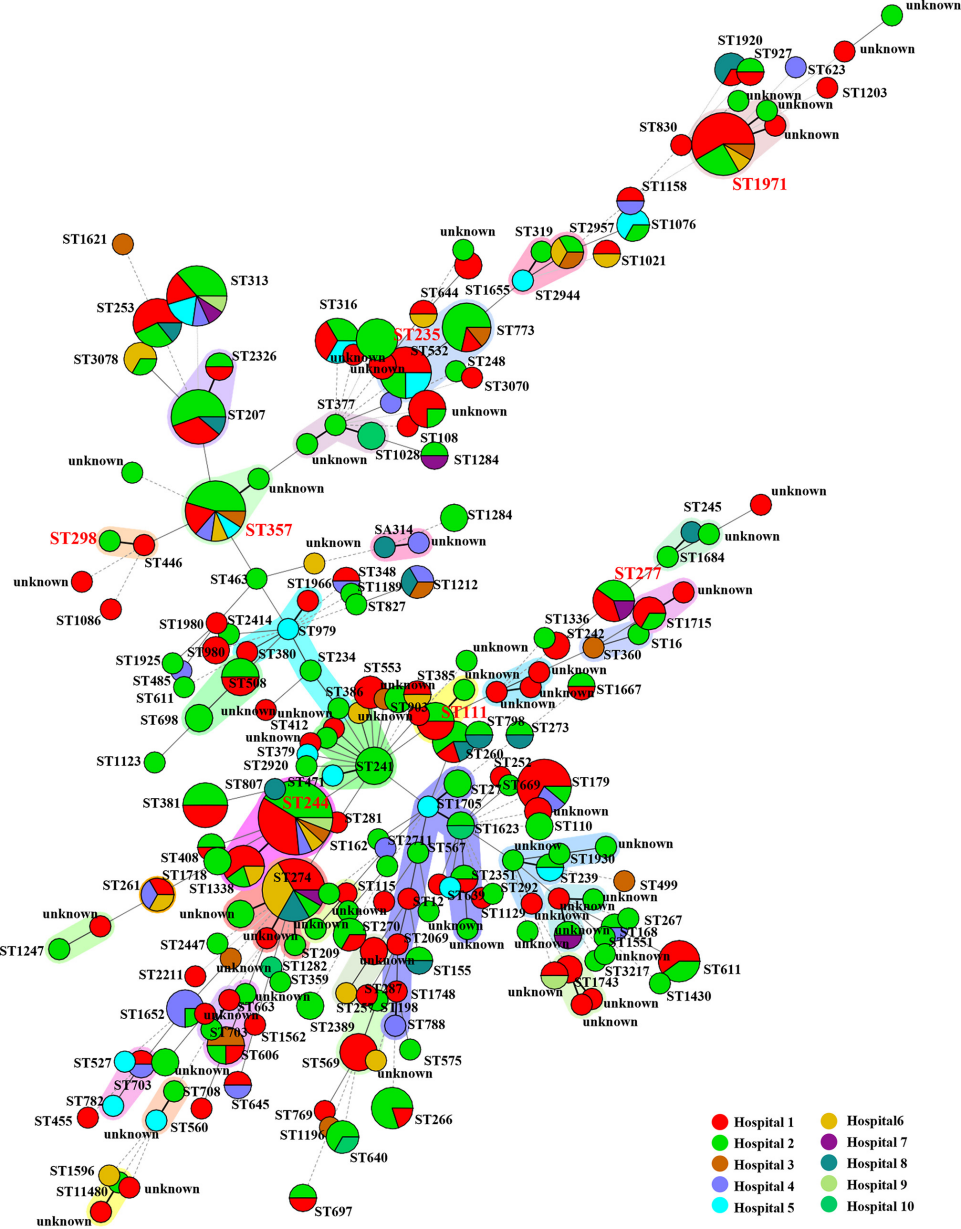
Geographical distribution of MLST types among 416 clinical P. aeruginosa strains collected from 10 tertiary hospitals in Guangdong, China, from 2017 to 2020. A total of 149 known STs and 72 new STs of strains were identified among these strains. The most prevalent STs were ST244 (*n =* 17), ST274 (*n =* 13), ST1971 (*n =* 12), ST313 (*n =* 11), ST357 (*n =* 11), ST179 (*n =* 9), ST532 (*n =* 8), ST773 (*n =* 7), ST253 (*n =* 7), ST316 (*n =* 6), ST277 (*n =* 5), and ST606 (*n =* 4). The high-risk STs are depicted in red text. The shading represents the observed clonal complex.

**FIG 3:**
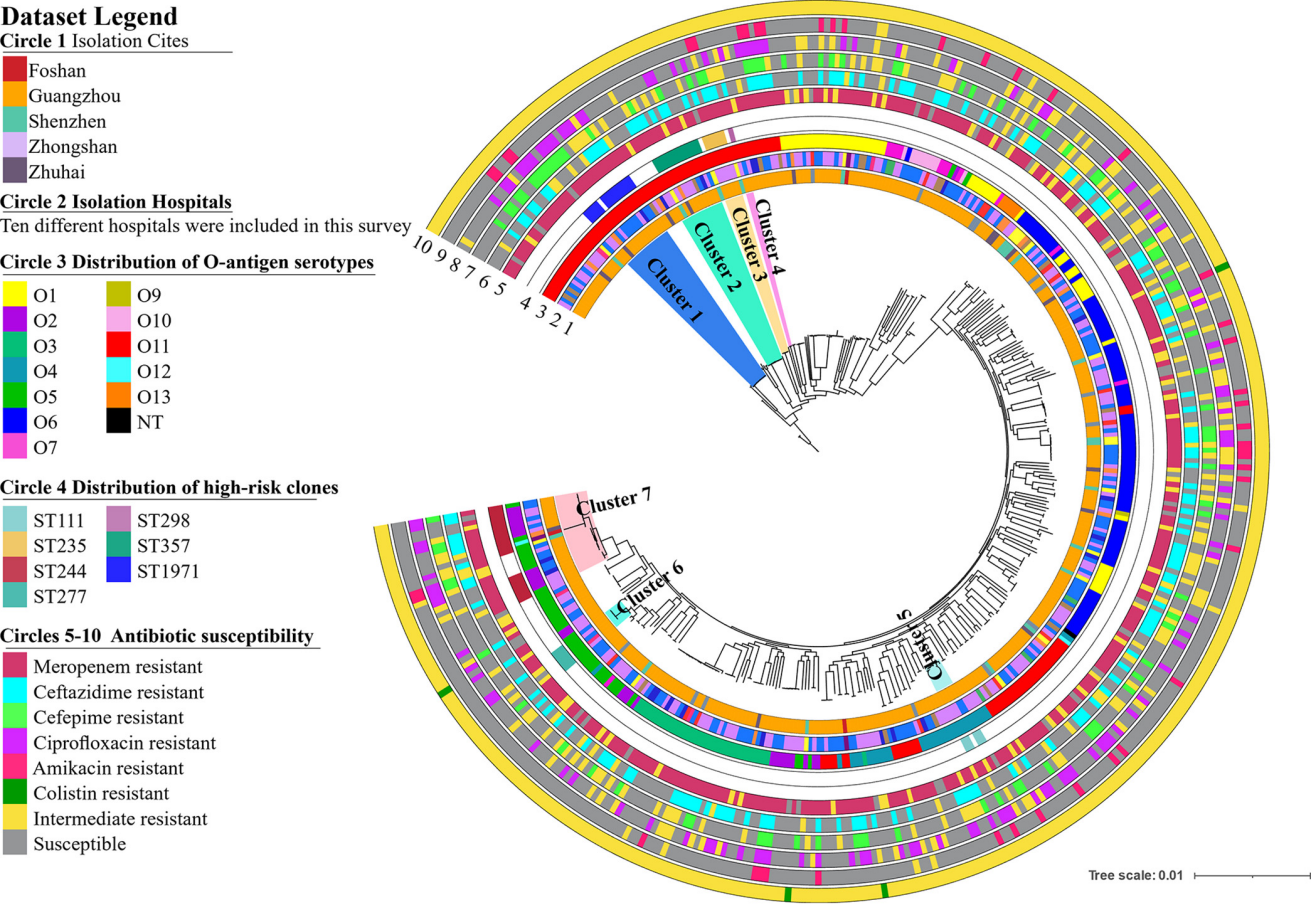
Phylogenetic tree of 416 P. aeruginosa strains collected from 10 tertiary hospitals in Guangdong, China, from 2017 to 2020. Circle 1 depicts strains isolated from five municipal cities in Guangdong, China. Circle 2 denotes 10 different investigating hospitals. Circle 3 depicts the distribution of O-antigen serotypes. Circle 4 depicts the distribution of high-risk clone strains. Circles 5 to 10 depict the antibiotic susceptibilities to currently used antibiotics.

**FIG 4:**
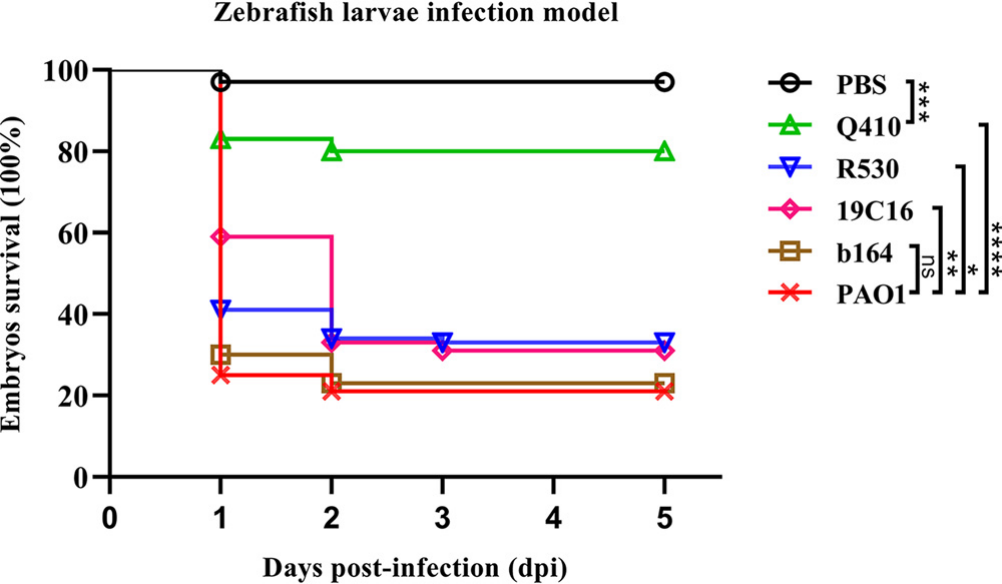
Virulence potential of the HiRiC ST1971 strains. Virulence potential of six strains, including the HiRiC ST1971 strains b164, R530, and 19C16, ST381 types of strain Q410, and control strain PAO1, were evaluated in a zebrafish larva infection model (*n* = 100). The survival rates of zebrafish larvae infected by corresponding strains (400 CFU) are presented in various colors. Statistical analysis was performed by using a log-rank (Mantel-Cox) test and GraphPad Prism 8.0. ****, *P < *0.0001; ***, *P = *0.0002; **, *P = *0.0058; *, *P = *0.0428; ns, *P = *0.6681.

To investigate the genetic characteristics of the epidemic CRPA strains recovered from hospital settings in Guangdong Province, WGS analysis was performed on the 416 P. aeruginosa strains. Multilocus sequence typing (MLST) analysis revealed that these strains exhibited a diversity of STs; a total of 149 known STs and 72 novel STs were identified, but none of these strain STs was predominant (Fig. 2). The core genes of these strains were relatively conserved. A total of 4,314 core genes were identified, accounting for nearly two-thirds of the total number of genes in each strain (Fig. 1h). The pangenome of these strains consisted of 46,757 genes and exhibited diverse genetic traits, which may be responsible for the high adaptability of these strains under different environments (Fig. 1g). The top 10 STs of the P. aeruginosa strains, as shown in Fig. 1e, accounted for a quarter of all test strains. The most commonly identified STs were ST244 (*n *= 17, 4.1%), ST274 (*n *= 13, 3.1%), and ST1971 (*n *= 12, 2.9%). A total of six known global HiRiCs STs (ST111, ST235, ST244, ST277, ST298, and ST357) and a novel HiRiC ST (ST1971) were detectable, three of which belonged to the top 10 STs (Fig. 1e). A phylogenetic tree was constructed to depict the genetic relatedness of the 416 P. aeruginosa strains based on the single nucleotide polymorphism (SNP) distance. Three STs—namely, ST1338, ST446, and ST3405—could be detected in the HiRiC clades, indicating that such strains pose a higher risk of infection. Cluster analysis also showed that clinical infections are caused by a wide variety of P. aeruginosa strains and that bacterial drug resistance occurs randomly and is not due to the transmission of specific clonal groups (Fig. 3). In particular, most strains among these six global HiRiC STs displayed large pairwise SNP distances in each clade and were confirmed to be the nonclonally disseminated colonies, despite the fact that they were recovered from the same clinical setting (see Tables S2 to S7 in the supplemental material). In contrast, ST1971 samples collected from two hospitals were found to display fewer differences in SNPs compared to strains of the other STs, with a distance of only 100 to 200 from each other (see Table S8). SNP correlation analysis showed that nearly one-third of mutations were missense variants, which were associated with genes that encode transcriptional regulators, membrane transporters, and various enzymes (see Table S9), rather than hypermutation genes relating to the formulation of drug resistance (16) (see Table S11). Importantly, genetic structure analysis revealed that an ~45-kb exogenous fragment was found to insert into the chromosome of ST1971 strains, which contained an ~25-kb multiple antibiotic-resistant island (ARI) upstream gene encoding ribonucleoside-diphosphate reductase subunit α. One integrase-encoding gene was also observed in the initial site of the exogenous region, which may then be the key factor of the formation of ARI. The genetic environments of corresponding resistant profiles were IS*26*-*aph(3′)-la*-IS*26* (Tn*6023*-like), IS*26*-*tet*(*C*)-*tetR*-IS*26* (Tn6309) and *aac(6′)lb*-*cmlA5*-*aadA2*-*drfA*-*qacE△1*-*sul1*, conferring resistance to four categories of antimicrobial agents, including sulfonamides, aminoglycosides, tetracyclines, and quinolones (see Fig. S2). The MIC data for ST1971 strains showed that 83.3% (10/12) of the strains displayed resistance to all Clinical and Laboratory Standards Institute (CLSI)-recommended antimicrobials tested except colistin and were regarded as XDR organisms. In the zebrafish larva model, infection of zebrafish embryos with 4 × 10^2^ CFU of HiRiC ST1971 strains, including b164, 19C16, and R530, led to 67 to 77% mortality at 2 days postinfection, which was very similar to that of high-virulence control PAO1, for which 79% mortality was obtained at 2 days postinfection (Fig. 4). For the negative-control and low-virulence groups, 97 and 80% survival rates were observed upon inoculation of saline and strain Q410 belonging to ST381. The MDR phenotype and the high transmissibility of such strains infer that they pose a serious threat of causing outbreaks in hospitals.

Here, strains of 12 serotypes that contained various O antigens or O polysaccharides have been found (Fig. 1f), with those carrying the antigens O11, O6, O1, and O5 being the most prevalent, accounting for 26.2, 20.4,12.7, and 9.9% of all O-antigen-bearing strains, respectively. O11 could be detected in four HiRiC STs of strains. Most of the strains with the same STs belonged to the same serotype, even though some of these strains were recovered from different hospitals. On the other hand, strains of the global HiRiC ST244 type of strains exhibited genetic differences, with three different O antigens identified among such strains (O2, *n *= 12; O5, *n *= 4; and O12, *n *= 1). Two different exotoxins, ExoU and ExoS, secreted by the type 3 secretion system in P. aeruginosa, exhibit a close relationship with bacterial serotypes, in which ExoU-secreting strains were frequently associated with O11, whereas O6 type strains were ExoU negative (17, 18). Our tests showed that 29.3% of the strains (122/416) belonged to O1, O11, O5, O7, and O10 serotypes and produced ExoU and that 69.5% of strains (289/416) of all test serotypes, except O12, secreted the exotoxin ExoS. Normally, ExoU and ExoS are usually not secreted by the same strain, yet in this study nine strains that expressed both ExoU and ExoS were identified. These nine strains were serotyped as O2 (*n* = 1), O3 (*n* = 2), and O4 (*n* = 6) and belonged to seven different STs. The effects of these two proteins on mediating infection in human need to be further investigated.

### Characterization of resistance profiles in CRPA isolates.

**FIG 5:**
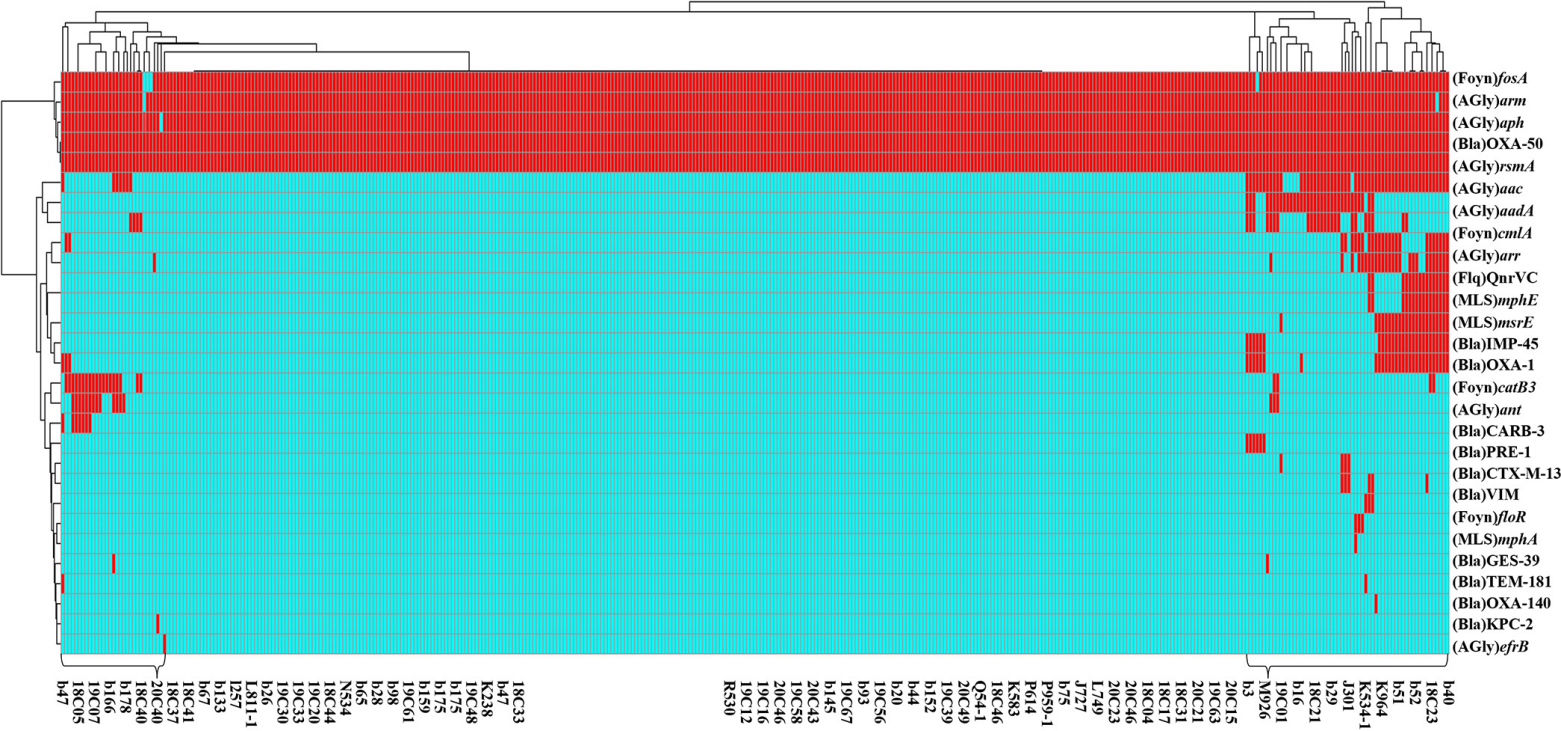
AMR gene analysis of 416 clinical P. aeruginosa strains collected in 10 tertiary hospitals in Guangdong, China, from 2017 to 2020. Heatmaps were obtained by aligning the draft genome sequence of each strain to the AMR gene database. P. aeruginosa strains are clustered using a maximum-likelihood tree. Red and cyan blue depict the presence and absence, respectively, of AMR genes in the test strains. The right side denotes the category of AMR genes. Almost all strains contained intrinsic AMR genes, including *fosA*, *armA*, *aph*, *bla*_OXA-50_, and *rsmA*. Only 17.8% (74/416) of the strains had acquired exogenous AMR genes. Strain names are depicted at the bottom of the chart.

A total of 34 antimicrobial resistance (AMR) genes were identified among the 416 CRPA isolates. These genes encoded resistance to eight categories of antimicrobial agents (Fig. 5). Five of them—namely, *fosA*, *armA*, *aph*, *bla*_OXA-50_, and *rsmA*—are intrinsic AMR genes that can be found in all test strains. Up to 77.2% (321/416) of the strains were found to be genetically conserved and only harbor these resistance genes in the chromosome. The other 22 exogenous AMR genes were sporadically distributed among the rest of the 74 strains, which accounted for only 17.8% (74/416) of the test strains. However, carriage of these exogenous AMR genes rendered these strains MDR. The 10 most prevalent exogenous AMR genes in these P. aeruginosa strains were *aac* (14.7%), *aadA* (8.2%), *catB3* (7.7%), *cmlA* (6.7%), *bla*_OXA-1_ (7.7%), *qnrVC* (6.5%), and *aar* (6.0%), which conferred resistance to six categories of antimicrobial agents, including aminoglycosides, phenicol, β-lactams, and quinolones. Lastly, six carbapenemase-encoding genes were identified. These genes were *bla*_IMP-45_ (*n* = 23, 5.5%), *bla*_CARB-3_ (*n* = 16, 3.8%), *bla*_PER-1_ (*n* = 7, 1.7%), *bla*_VIM_ (*n* = 6, 1.4%), *bla*_GES-39_ (*n* = 2, 0.5%), and *bla*_KPC-2_ (*n* = 1, 0.2%). They were normally plasmid-borne and hence highly transmissible, conferring carbapenem resistance among clinical strains.

### Mechanisms of carbapenem antibiotic resistance in clinical CRPA isolates.

WGS analysis showed that a total of 13.0% (54/416) of the P. aeruginosa strains tested in this study harbored carbapenemase-encoding genes and consistently exhibiting meropenem resistance. Among the 54 meropenem-resistant strains, one strain that carried *bla*_KPC-2_, two strains that carried *bla*_GES-39_, and six strains that carried *bla*_VIM_ gene were resistant to imipeneml however, 7 of the 23 *bla*_IMP-45_-carrying strains, 2 of the 16 *bla*_CARB-3_-carrying strains, and 2 of the 7 *bla*_PER-1_-carrying strains were sensitive to imipenem, suggesting that these genes were not the key factors mediating their imipenem resistance. Carbapenem resistance in other strains may be caused by inactivation of the *oprD* gene (imipenem resistance) or overexpression of efflux genes, including those encoding the MexAB-OprM, MexCD-OprJ, and MexXY-OprM pumps (meropenem resistance). BLASTP analysis showed that the *oprD* gene commonly harbors mutational changes, with those exhibiting an indel pattern being the most common and observable in 86.8% (361/416) of the test strains. On the other hand, 39 strains (9.4%) were found to contain missense mutations; only 16 (3.8%) strains exhibited 100% homology with the P. aeruginosa strain PAO1. However, carriage of *oprD* mutations did not always correlate with expression of the imipenem resistance phenotype. To investigate whether mutational changes in the *oprD* genes are the underlying mechanism of expression of the carbapenem resistance phenotype, we analyzed the BLASTP results and examined the relationship between different types of mutations and the carbapenem susceptibilities of the test strains. Strains were allocated into two categories according to the types of *oprD* mutations that they harbored. The first class involved strains in which the *oprD* gene had a frameshift caused by insertion sequence and those which exhibited an indel pattern. Up to 48.01% (193/402) of CRPA strains belonged to this class, all of which exhibited imipenem resistance. The second class of strains contained wild-type sequence or mutated/indel pattern without causing frameshift in protein-encoding sequence. A total of 209 strains belonged to this group and their *oprD* gene were found to exhibit 92.5 to 100% sequence homology with the wild-type gene. Interestingly, most of the strains which contained homologous *oprD* sequences in this group shared identical target mutations, except for those which have acquired a new stop codon; among these strains, a total of 16 mutation types were identified (Table 2). We found that mutations that created a stop codon in the *oprD* gene was a key mechanism that mediates the onset of imipenem resistance. As many as 45.0% (94/209) of the strains in group 2 were found to contain a stop codon in this gene. The stop codon was also frequently detected in some strains carrying identical mutation patterns. Further analysis indicates that a total of 29 different mutations that resulted in formation of a stop codon in the *oprD* gene among these strains could be assigned to 12 *oprD* mutation combination groups, of which group 6 was found to have six different stop codon target sites, with a detection rate of 88.6% (39/44). In addition, some strains carrying identical mutations did not exhibit the same imipenem susceptibility phenotype, suggesting that such mutations did not contribute to imipenem resistance. BLASTP analysis confirmed that a total of 115 strains did not contain stop codon mutations in the *oprD* gene, and yet 90 of these strains exhibited resistance to imipenem. Target mutations in these strains could be assigned to 12 mutation combinations, and yet a correlation between these mutations and imipenem resistance was not observed. Seven mutation types detectable in 80 strains were found to be susceptible to imipenem resistance; consistently, imipenem-sensitive strains that carried such mutations were identified. In the seventh mutation group, the *oprD* gene in 35 strains exhibited 94.6% identity to the wild-type gene and shared 24 identical mutation sites, but four of these strains were sensitive to imipenem. To further confirm the relationship between mutated *oprD* gene and imipenem resistance, a pair of isogenic strains in this mutation group, i.e., 18C53 (imipenem resistance, MIC > 16) and 18C54 (imipenem susceptible, MIC = 1), which belonged to ST1401, exhibited 42 SNP differences, and did not contain any carbapenemase-encoding genes, were selected to assess the expression level of the *oprD* gene to investigate the degree of correlation with the expression level of the *oprD* gene and imipenem resistance. The difference in the expression level of the *oprD* gene in the two isogenic strains was minimal, indicating that the imipenem resistance phenotype of one of the strains was not due to this type of mutation (see Fig. S3). The mechanisms of resistance to imipenem in such strains merits further investigation.

**TABLE 2:** Correlation analysis of *oprD* mutations (noninsertion and frameshift) and IMP resistance in P. aeruginosa

Mutation type	No. of strains	No. of strains mutated to stop codons (IMP resistance)	Association of *oprD*-bearing strains without stop codon mutations and drug resistance phenotypes^*a*^			
			No. of strains without stop codon mutations	No. of core/total no. of target mutations	Resistance rates (%) of strains without stop codon mutations	Correlation with resistance
1	1	1	0	–	–	–
2	5	4	1	31/31	100 (1/1)	
3	22	11	11	26/30	91 (10/11)	NA
4	10	0	10	29/29	70 (7/10)	NA
5	1	1	0	–	–	–
6	44	39	5	22/25	100 (5/5)	
7	37	2	35	24/24	88.6 (31/35)	NA
8	4	3	1	11/11	100 (1/1)	
9	13	9	4	10/10	25 (1/4)	NA
10	14	0	14	9/9	64.2 (9/14)	NA
11	6	6	0	–	–	–
12	12	6	6	3/4	66.7 (4/6)	NA
13	12	2	10	3/3	70 (7/10)	NA
14	4	4	0	–	–	–
15	8	6	2	0/1	100 (2/2)	
16	16	0	16	0/0	75 (12/16)	NA
Total	209	94	115	–	78.2 (90/115)	

a NA, strains with identical mutation sites exhibited different imipenem-resistant phenotypes, indicating that the mutation type does not correlate with imipenem resistance. –, No strains met the criteria for *oprD*-bearing strains without stop codon mutations and drug resistance phenotypes.

Meropenem resistance mechanisms in the 416 clinical P. aeruginosa strains were also investigated. Dissemination of mobile carbapenem resistance determinants (13.1%) was apparently responsible for the increased prevalence of meropenem resistance, with *bla*_IPM-45_ (*n* = 23, 5.5%) being the most common resistance gene that was able to confer high-level resistance (MIC >16) to meropenem. On the other hand, three efflux systems, including MexAB-OprM, MexCD-OprJ, and MexXY-OprM, have been reported to be closely associated with the expression of meropenem resistance in clinical P. aeruginosa strains. To better understand the underlying molecular mechanisms, BLASTP analysis were performed on these three efflux-pump-encoding clusters and the related regulatory genes. For the MexAB-OprM efflux pump systems (19), the mutation rates of *mexA*, *mexB*, and *oprM* genes were 9.1, 21.9, and 13.2%, respectively, but most of the target gene mutations were not associated with drug resistance, since meropenem-susceptible strains were also found to contain the same mutations in these genes. The regulatory genes of this efflux system include *mexR*, *nalC*, and *nalD*. Up to 57.7% (240/416) of the test strains were found to contain different *mexR* variants. Obviously, the common mutation *mexR*^V126E^ did not cause the overexpression of MexAB-OprM and meropenem resistance. Yet the remaining 118 variants (28.4%) exhibited meropenem resistance, especially those harboring the following five types of mutational changes in the *mexR* gene: (i) stop codon mutations (*n* = 12), (ii) the insertion of a large fragment with or without frameshift (*n* = 43, two different sizes of *ΔmerR* in same contig), (iii) truncation (*n* = 5, two different sizes of *ΔmerR* in separate contigs), (iv) deletion resulting in the loss of specific amino acid-encoding codons (*n* = 5), and (v) mutation excluding *mexR*^V126E^, a total of 27 different mutation types were recoverable from 47 strains, 37 of which exhibited resistance to meropenem, the remaining 10 exhibited intermediate resistance to meropenem. The most prevalent mutation among these strains was *mexR*^T130P^ (*n* = 11), followed by *mexR*^A103G^ (*n* = 6) and *mexR*^D89G^ (*n* = 3). In contrast, the *nalC* gene, a positive regulator of MexAB-OprM, was found to exhibit a high mutation rate (89.66%, *n *= 373), but only three types of variants, namely, *nalC*^S209R, G71E^ (*n* = 291) *nalC*^G71E^ (*n* = 41), and *nalC*^A186T, G71E^ (*n* = 34), were identified. Based on the resistance phenotypes of these bacteria, these mutations do not appear to be responsible for causing meropenem resistance. In addition, the *nalD* gene is relatively conserved, with only 13.7% of strains (57/416) found to harbor mutations in this gene. Nevertheless, missense and frameshift mutations were both detectable in this gene. A total of 16 different target mutations could be detected in 30 strains, the prevalent ones being *nalD*^G206S^ (*n* = 7), *nalD*^T188A^ (*n* = 6), and *nalD*^D187H^ (*n* = 4). Importantly, all of these mutants exhibited resistance to meropenem, except for three strains carrying the mutations *nalD*^D147E^, *nalD*^Q114^* (“*” stands for stop codon), and *nalD*^Q35^*. Strains in which the *nalD* was inactivated by frameshift mutations (*n* = 19, four-base deletion), there was an insertion by a large fragment (*n* = 1), or there was an amino acid deletion (*n* = 2) were all resistant to meropenem, suggesting that the regulatory gene *nalD* plays a key role in mediating expression of the *mexAB-oprM* systems and hence meropenem resistance. Interesting, *mexR* and *nalD*-mediated meropenem resistance mechanisms are independent; we estimated that the MexAB-OprM system in 53.2% (164/308) of meropenem resistant strains was overexpressed due to positive regulation by the *nalD* gene.

The two other efflux-pump-encoding genes, namely, the MexCD-OprJ and MexXY-OprM operons, and the corresponding regulatory repressor genes *nfxB*, *esrC*, and *armR* were also subjected to alignment analysis. Mutation profiles in the MexCD-OprJ cluster were highly diverse, whereas the regulatory genes were relatively conserved. Mutation rates of 79.6% (331/416), 99.0% (412/416), and 74.3% (309/416) in the *mexC*, *mexD*, and *oprJ* genes, respectively, were recorded. In the regulatory genes *nfxB* and *esrC*, the mutation rates were 10.8% (45/416) and 32.0% (133/416), respectively. Furthermore, 51, 47, and 35 types of mutation combinations were observable in the *mexC*, *mexD*, and *oprJ* genes, respectively. The target mutation combinations in meropenem-sensitive and -resistant strains were compared. We found that 86.1% (285/331) of mutations including 26 mutation combinations in *mexC*, 90.8% (374/412) of mutations including 32 mutation combinations in *mexD*, and 91.9% (284/309) of mutations involving 18 mutation combinations in *oprJ* were not regarded as being responsible for meropenem resistance, since the same mutations were observed in meropenem-sensitive strains. Likewise, 88.9% (40/45) and 81.2% (108/133) of mutations in *nfxB* and *esrC* genes, respectively, were deemed not responsible for causing meropenem resistance. In addition, we observed that insertions and deletions in the *mexD* gene conferred meropenem resistance, but the incidence of such events was extremely low, with only four such strains being detected. The remaining 27.9% (86/308) meropenem-resistant strains were found to harbor at least one mutation in these MexCD-OprJ efflux pump genes, but whether these mutations play a role in the expression of meropenem resistance cannot be confirmed. Apart from strains that exuding meropenem through the MexAB-OprM system, we estimated that at least 17.2% (53/308) of the strains developed meropenem resistance by harboring an activated MexCD-OprJ efflux pump system. In the MexXY-OprM gene cluster, the incidences of mutation and insertion events in *mexX* and *mexY* genes were 99.3% (413/416) and 98.6% (410/416), respectively, whereas the proportion of such events in upstream regulatory *armR* genes was 37.0% (154/416). Our data showed that the incidence of occurrence of missense mutation and truncation in the *armR* gene was 25.2% (105/416) and 11.8% (49/416), respectively. The meropenem resistance rates in strains carrying an *armR* variant (73.4%, 113/154) was much higher than strains (53.5%, 139/260) harboring the wild type *armR* gene, indicating that the *armR* gene in the MexXY-OprM system is more closely associated with meropenem resistance than the *mexX* and *mexY* genes. A total of 37 types of *armR* mutations variants were detectable in 108 strains, most of which were single and double mutants. Among them, 14 types of *armR* variants were not associated with meropenem; these *armR* mutations, in a total of 63 strains, including the most prevalent *armR*^L138R^ mutation (*n* = 45), were therefore not regarded as being able to activate the OprXY-OprM systems and confer meropenem resistance in the host strain. At the same time, 13 strains harboring a truncated *armR* gene due to insertion of exogenous genetic sequences were sensitive to meropenem, suggesting that *armR* does not play a key role in development of meropenem resistance through activation of the MexXY-OprM system. Furthermore, correlation analysis of carbapenems resistance and the chromosomal cephalosporinase *ampC* genes was also performed in this study, with a total of 78 kinds of variants being identified, among which PDC-423, PDC-442, and PDC-24 were the most prevalent, with 23, 22, and 23 cases being detected, respectively. However, no *ampC* variants were found to be associated with carbapenem resistance.

## DISCUSSION

The incidence of CRPA infections has significantly increased worldwide and become a serious public health issue. In a 4-year surveillance program conducted in Guangdong Province, China, we collected 416 clinical P. aeruginosa strains from 10 tertiary hospitals, 96.9% of which were found to belong to CRPA strains. Importantly, as many as 64.8 and 15.7% of these CRPA strains could be categorized as MDR and XDR. Genetic characterization of these CRPA strains showed that only 13.1% of the strains carried MBL-encoding genes that exhibited high carbapenem-resistant potential. A previous study had shown that the proportion of P. aeruginosa strains that harbored carbapenem resistance determinants increased from 12.3% in 2010 to 30.6% in 2011 in 14 European countries (11), whereas we showed here that imipenem and meropenem resistance in these strains was mainly due to mutations in the porin D gene *oprD*, as well as to the overexpression of specific efflux pumps, as previously reported (20). In particular, we confirmed that mutational changes in *oprD* was strongly associated with imipenem resistance. Such changes include complete inactivation of the *oprD* gene by frameshift mutation, insertion of a large fragment, and mutations that lead to the creation of a stop codon but not missense mutations. Likewise, mutations in the *mexAB* and *oprM* genes of the MexAB-OprM efflux systems were less closely associated with meropenem resistance; their regulatory genes, *mexR* and *nalD*, however, appear to play a critical role in mediating development of meropenem resistance, since mutations in these two regulatory genes were found in 53.2% of the 308 meropenem-resistant strains. In addition, we found that the single mutation *mexR*^V126E^ did not confer meropenem resistance. Amsalu et al. also reported that 15 strains carrying such *mexR* mutations exhibited 1.896- to 5.911-fold expression of the MexA pump compared to those carrying the wild-type sequence, but these strains exhibited varied susceptibility to meropenem compared to the wild-type PAO1 strains (21). In the MexCD-OprJ efflux pump system, the effects of mutational changes in efflux-pump-encoding genes and the corresponding regulatory genes on drug susceptibility are highly complex, and a firm conclusion could not be made based on existing data. However, we can confirm herey that meropenem resistance was not strongly associated with the activities of the efflux-pump-encoding genes in the MexCD-OprJ system. Although a high mutation rate was detectable in these genes, most of these mutations could be observed in both meropenem-resistant and -susceptible strains. Mutations in these efflux-pump-encoding and regulatory genes that were uniquely present in 27.8% of all the meropenem-resistant strains. It is likely that insertions and deletions in the *mexD* gene may result in meropenem resistance, but only an extremely small proportion of resistant strains have such mutational changes. Excluding the effect of MexAB-OprM, only 17.2% of meropenem-resistant bacteria exhibited overexpression of the MexCD-OprJ pump. For the *mexXY-oprM* system, mutations can be found in the *mexX* and *mexY* genes of almost all the test strains, so that correlation between such mutations and meropenem resistance cannot be established. In addition, mutations in the regulatory gene *armR* may also exhibit weak relationship to meropenem resistance. Among the 35 types (accounting for 37.3% [155/416] of all test strains) of *armR* variants observed, 7 types (accounting for 21.9% [91/416] of all test strains) can be detected in both meropenem-resistant and -susceptible strains. There was no obvious increase in the resistance rate of meropenem (73.4%) in strains that contained *armR* variants, for which the proportion of meropenem-resistant strains in this surveillance was 74.0%, as described above.

Findings of this work also confirm the epidemiological characteristics of clinical CRPA strains collected from Guangdong, China. MLST analysis showed that various STs of P. aeruginosa strains could cause infections in clinical settings. On one hand, their core genes are particularly conserved. Nearly two-thirds of the genes can be found in over 200 ST-tested strains; these genes are known to be responsible for encoding broad-spectrum pathogenicity and high prevalence of P. aeruginosa strains in this region. The situation is aggravated by the emergence of MBL-producing and HiRiC strains. HiRiC isolates are known to carry MBL encoding genes and have been widely disseminated around the world (22, 23). Here, we not only detected strains of six global HiRiCs STs but also found that 22.8% of the test strains harbored exogenous AMR genes. We also identified 25 STs of strains that carried MBL determinants. Dissemination of MDR-encoding plasmids was a common event, among which a plasmid named p243931-IMP (accession number MN208062), which harbored the *bla*_IMP-45_ gene, was observed in 14 nonclonal strains of eight different STs that have disseminated extensively. In addition, a unique HiRiC ST (ST1971) in China and three ST strains, namely, ST1338, ST446, and ST3405, the genetic contents of which were similar to those of HiRiC strains, were identified. In particular, the ST1971 strains exhibited an XDR phenotype and high genetic correlations and displayed high virulence as PAO1 in a zebrafish infection model, indicating that this strain ST can cause infections and does not respond well to the majority of commonly used antimicrobial agents. HiRiC ST179 carrying *qnrVC1* and *bla*_IMP-25_ genes was another problematic clone that can transfer resistance elements to other organisms. On the other hand, specific serotypes were also found to be strongly associated with bacterial pathogenesis. Currently, a total of 20 different O serotypes were identified, among which O1, O2, O5, O16, O6, and O11 serotypes have been reported to be responsible for 70% of P. aeruginosa infections (17). Importantly, 76.4% of strains in this study belonged to these highly pathogenic serotypes, highlighting the public health implications of these pathogenic clones. P. aeruginosa is known to inject toxic effector substances such as ExoU, ExoS, ExoT, and ExoY into the cytoplasm of eukaryotic cells through the type 3 secretion system, enhancing the severity of infection (24, 25). In a mouse model established for the investigation of bacterium-mediated acute pneumonia and the related mortality rate, as well as dissemination and persistence of the bacteria in the lungs, ExoU-secreting strains were determined to be significantly more virulent than ExoS-secreting strains (26). Surveillance data showed that among our test strains, 69.5% contained *exoS* and 29.3% contained *exoU*; this ratio is similar to that of a large survey of clinical strains performed two decades ago (27). Nevertheless, nine strains carrying both *exoU* and *exoS* genes were observed in this study, so further investigation on the impact of this newly evolved clone on the virulence level in P. aeruginosa is necessary.

In conclusion, this study reported the results of a large-scale genetic surveillance and molecular characterization program of CRPA strains in Guangdong, China. To date, CRPA infection remains a serious public health issue worldwide. Control measures, such as impeding the dissemination and outbreak of CRPA isolates, should be devised, along with the development of inhibitors targeting efflux-pump-encoding genes. Surveillance on the evolutionary and epidemiological characteristics of clinical CRPA strains is also necessary to improve the accuracy of clinical diagnosis and to provide updated knowledge that enhances the effectiveness of infection control measures designed to reduce the incidence and mortality of CRPA infections.

## MATERIALS AND METHODS

### Bacterial strains and species identification.

A total of 416 P. aeruginosa strains recovered from clinical specimens—including ascitic fluid, bile, blood, bronchoalveolar lavage fluid, hydrothorax, lumbar puncture, secretions, sputum, tissue, urine, and wound/abscess drainage samples—were collected from 29 clinical departments in 10 tertiary hospitals in Guangdong province, China, from 2017 to 2020. The antimicrobial susceptibility of the strains was determined by the broth microdilution method according to CLSI guidelines, with Escherichia coli ATCC 25922 and Staphylococcus aureus ATCC 25923 being used as the quality control strains (28, 29). A polychoric correlation test was performed to measure the correlation coefficient between the age and antibiotic resistance level using the R package Psych.

### Whole-genome sequencing and analysis.

Genomic DNA of the 416 P. aeruginosa strains were extracted using a MagAttract HMW DNA kit (Qiagen, Germany). DNA libraries were constructed by using a MGIEasy stLFR Library Prep kit v1.1 (PN: 1000005622), according to a modified procedure (30). The, 100-bp paired-end reads were obtained from the DNBSEQ platforms of the China National GenBank. Raw sequencing reads were trimmed and quality filtered by using Trimmomatic (v0.38) and FastQC v0.11.3 software, respectively, with default parameters. Draft genes in the genome were acquired by using Shovill v1.0.9, a software that uses SPAdes for genome assembly (31, 32). The assembled genome was then annotated using the Prokka v1.14.6 (33) and the NCBI Prokaryotic Genome Annotation Pipeline. Pan- and core-genome sizes were estimated by Roary (34) and visualized with the R package, using vegan (35) and ggplot2 (36). ST and serotype identifications of the test strains were confirmed by MLST (37) and PAst 1.0 based on the MLST database (http://github.com/tseemann/mlst) and the serotype database (https://cge.cbs.dtu.dk/services/PAst/), respectively. To detect AMR genes and determine the genetic characteristics of these genes in clinical P. aeruginosa strains, draft sequences searches were conducted by BLAST using the Comprehensive Antibiotic Resistance Database (CARD, https://card.mcmaster.ca/) (38).

### Phylogenetic analysis.

Trimmed and quality-filtered sequences obtained from the 416 P. aeruginosa strains were aligned to a CRPA isolate, namely, 18C01, which was initially collected in Guangzhou City in 2018. SNPs were identified by Snippy v3.1 with default settings (39), followed by short-read mapping by BWA-MEM v0.7.12. The alignment results were then filtered and used to generate two alignment files known as “core-aln” and “core-full-aln,” of which “core-full-aln” was subjected to ML phylogenetic analysis using iqtree v1.6.12 (40) and under the GTR+F+R10 model. The phylogenetic tree was graphically visualized by iTOL version 3 (41). The MLST analysis results, which identified 149 known and 72 novel MLST strains, were also subjected to clustering and SNP distance analysis.

### Mutation analysis.

Gene sequences encoding the reference proteins OprD and the MexAB-OprM, MexCD-OprJ, and MexXY-OprM efflux pumps in P. aeruginosa strain PAO1 (accession number NC_002516) were obtained and aligned with each target strain by BLASTp script. Alignment results, including the located contig, completeness of target gene alignment, the identity rate, and the start and stop positions of target genes, were recorded. The aligned sequences were grabbed by the “grep” script and integrated into a Fasta file according to the alignment record. The Molecular Evolutionary Genetic analysis software was used to identify the mutation site; the alignment record was analyzed by the CLC Genomics Workbench to confirm occurrence of amino acid indels, frameshift and large sequence insertion in the target genes.

### Quantitative real-time PCR assay.

To evaluate the degree of correlation between carriage of *oprD* mutations (non-stop codon mutation) and imipenem resistance, the expression level of the *oprD* gene in strains 18C53 and 18C54 was determined by qPCR. Total RNA of the two strains were extracted and purified by using the Qiagen RNeasy minikit and Turbo DNA free kit (Qiagen, Inc.). cDNA was synthesized using a SuperScript IV reverse transcriptase kit (Invitrogen), with *rplS* being used as a housekeeper gene to normalize the expression levels of the target genes. Primers and conditions used in qPCR are listed in (see Table S1). Quantitative real-time PCR assay was performed by using the QuantStudio 7 real-time PCR system (Applied Biosystems) and Qiagen SYBR green master mix (Qiagen, Inc.) The tests were performed in triplicates. The results of qRT-PCR were analyzed by the Design & Analysis Software 2.6.0 and visualized by GraphPad Prism v8.0.1.

### Zebrafish larvae infection model.

A zebrafish larvae infection model was used to access the virulence potential of HiRiC ST1971 strains as previous study (42). Zebrafish embryos at 30 h postfertilization were used in this study. A total of 100 embryos randomly assigned into each group were intravenously inoculated with 400 CFU of tested P. aeruginosa strains. Survival of zebrafish larvae was observed for 120 h postinfection at 24-h intervals. P. aeruginosa strain PAO1 and a low-virulence P. aeruginosa strain were used as controls.

### Ethics declarations.

Ethical permission was approved by the Ethics Committee of Zhujiang Hospital of Southern Medical University on 30 May 2021 under approval 2021-KY-046-01.

### Data availability.

The WGS data in this study were deposited in GenBank under BioProject accession number PRJNA832853. Details and individual accession numbers for sequence data included in our analyses are available in Table S10 in the supplemental material.
