# Supplementary material for: Epidemiological and Genetic Characteristics of Clinical Carbapenem-Resistant Pseudomonas aeruginosa Strains in Guangdong Province, China
Source: Microbiol Spectr. 2023 Apr 20;11(3):e04261-22. doi: 10.1128/spectrum.04261-22 (PMC10269565; doi:10.1128/spectrum.04261-22)
Supplement: Supplemental file 1 — Supplemental material. Download spectrum.04261-22-s0003.pdf, PDF file, 0.7 MB [file spectrum.04261-22-s0003.pdf]

Supplementary

Epidemiological and Genetic Characteristics of Clinical Carbapenem-Resistant *Pseudomonas aeruginosa* Strains in Guangdong Province, China

Yonggang Zhao[1, #], Dingqiang Chen[2, #], Kaichao Chen[3, #], Miaomiao Xie[3], Jiubiao Guo[4], Edward Wai Chi Chan[3], Lu Xie[5], Jingbo Wang[4], Enqi Chen[4], Sheng Chen[3*], Weijun Chen[6*], Lars Jelsbak[1*]

Running title: Epidemiological characteristics of clinical CRPA

[1]Department of Biotechnology and Biomedicine, Technical University of Denmark, Denmark.

[2]Microbiome Medicine Center, Department of Laboratory Medicine, Zhujiang Hospital, Southern Medical University, Guangzhou Guangdong, 510282, China.

[3]Department of Infectious Diseases and Public Health, Jockey Club College of Veterinary Medicine and Life Sciences, City University of Hong Kong, Kowloon, Hong Kong.

[4]College of Pharmacy-Shenzhen Technology University. Shenzhen Guangdong, 518118, China.

[5]Research Center for Micro-Ecological Agent Engineering and Technology of Guangdong Province, Guangzhou Guangdong, 510640, China.

[6]BGI-Shenzhen, Shenzhen Guangdong, 518083, China.

# These authors contributed equally to this work.

Corresponding author:

Sheng Chen, City University of Hong Kong. Email: shechen@cityu.edu.hk.

Weijun Chen, BGI-Shenzhen. Email: chenwj@bgi.com.

Lars Jelsbak, Technical University of Denmark. Email: lj@bio.dtu.dk.

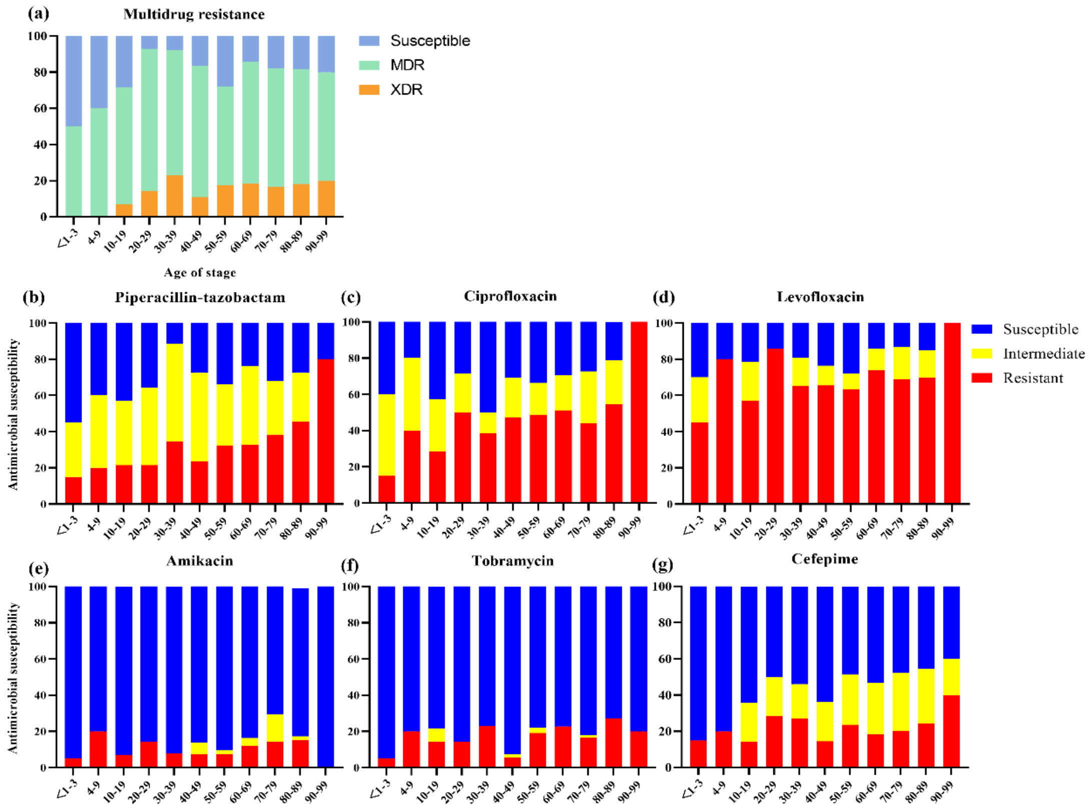

Supplementary Figure S1. Correlation of patient ages and antimicrobial susceptibility in 416 clinical *P. aeruginosa* strains. (a) Distribution of susceptible, MDR and XDR *P. aeruginosa* strains in patients of different ages. Susceptibility of piperacillin-tazobactam (b), ciprofloxacin (c), levofloxacin (d), amikacin (e), tobramycin (f) and cefepime (g) of the 416 *P. aeruginosa* strains from patients of different ages.

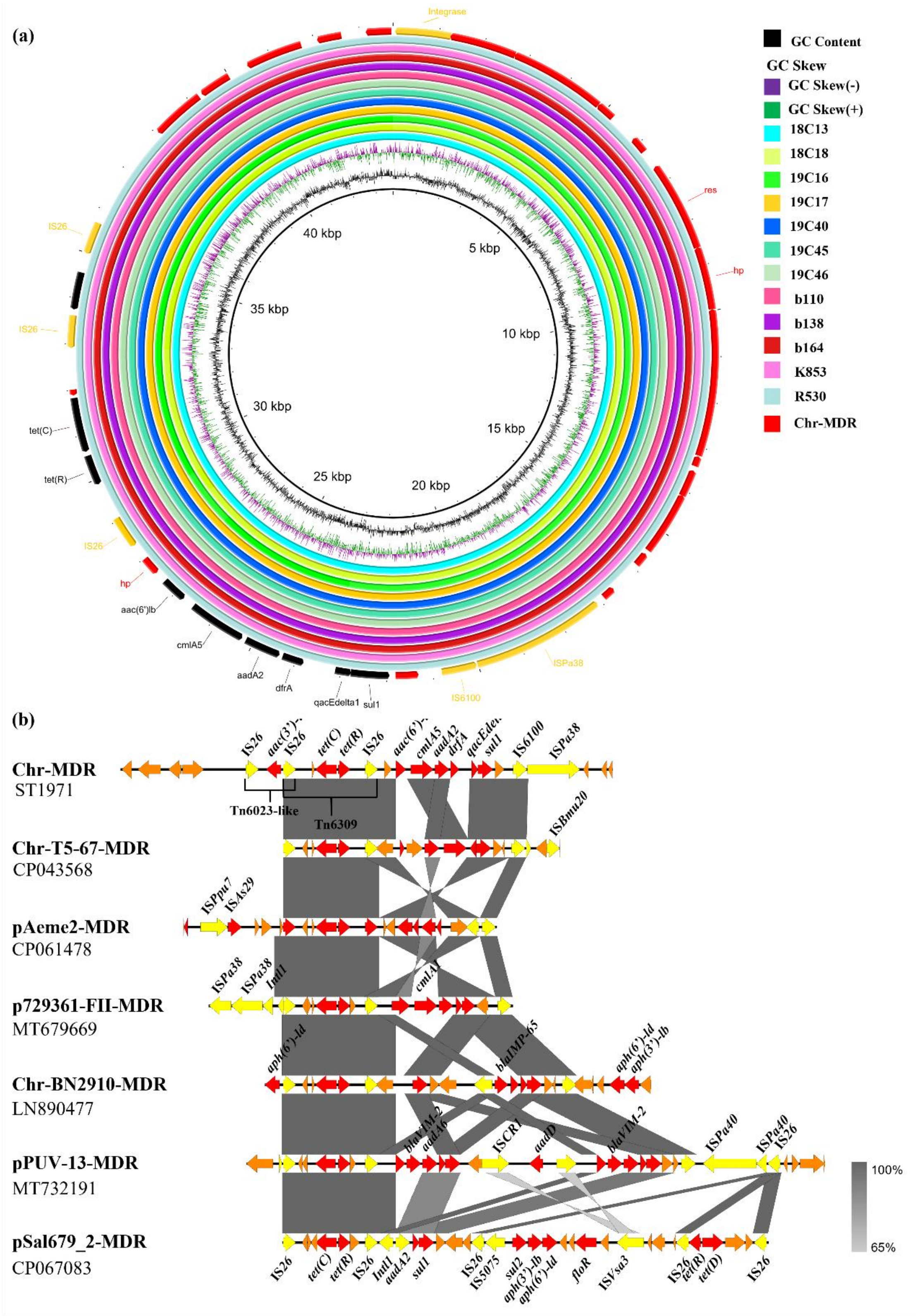

Supplementary Figure S2. Genetic structure of MDR region in ST1971 *P. aeruginosa* strains. (a) Circular alignment of MDR regions of strain R530 with ST1971 strains in this study, using Blast Ring Image Generator (BRIG). (b) Linear alignment of chromosomal antibiotic-resistant island from ST1971 strains with the

similar structures deposited in the NCBI database, including chromosomal MDR regions Chr-T5-67-MDR (CP043568) and Chr-BN2910 (LN890477) recovered from *Comamonas testosterone* and *Achromobacter xylosoxidans*, respectively; and MDR regions of plasmid pAeme2 (CP061478, *Aeromonas caviae*), plasmid p729361-FII (MT679669, *Leclercia sp*), plasmid pPUV-13 (MT732191, *Pseudomonas aeruginosa*) and pSal679_2 (CP067083.1, *Salmonella enterica subsp*). Red, resistance gene; yellow, insertion sequence.

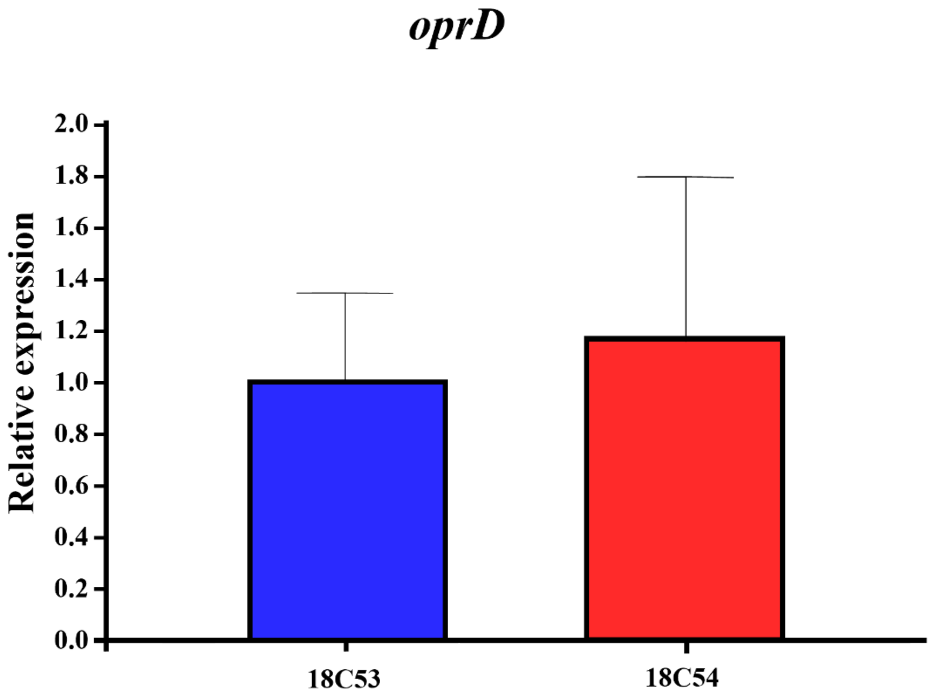

Supplementary Figure S3. Relative expression levels of *oprD* gene in strains 18C53(IMP-resistant) and 18C54 (IMP- susceptible). Slight expression differences with 1.2-fold were observed, the *rpls* gene were used as a housekeeper gene to normalize the expression levels of the target genes.
